# Metaphors in context and in isolation: Familiarity, aptness, concreteness, metaphoricity, and structure norms for 300 two-word expressions

**DOI:** 10.3758/s13428-025-02902-0

**Published:** 2026-01-05

**Authors:** Laura Pissani, Roberto G. de Almeida

**Affiliations:** 1 Department of Language Science and Technology, Language Science and Technology, Saarland University, Campus C7 2, Room 3.04, 66123 Saarbrücken, Germany; 2 Department of Psychology, Concordia University, Montreal, Canada

**Keywords:** Metaphors, Metaphor norms, Metaphors in context, Compositionality, Figurative language

## Abstract

Familiarity, aptness, concreteness, metaphoricity, and structural norms for 300 two-word English metaphorical expressions (e.g., *broken heart*, *early bird*), presented in sentence context and in isolation, were obtained from 164 participants. Familiarity was conceived as the extent to which participants had previously heard or read that expression. Aptness was conceived as the extent to which the vehicle captured important features of the topic. Concreteness was conceived as the extent to which the meaning conveyed by the vehicle could be perceived through the senses or actions. Metaphoricity was conceived as the extent to which the expression was perceived as figuratively rather than literally true. Metaphor constituent structure was conceived as a graded measure indicating whether the metaphorical content is carried by the first word, the second word, or distributed across both words. In addition to these variables, which are known to play a key role in metaphor comprehension, we provide frequency scores for the whole expression as well as for each constituent separately from the Corpus of Contemporary American English (COCA) database. Cumulative link mixed-effects models were used to examine the effects of context and vehicle position on participants’ ratings, and to assess whether familiarity, aptness, and concreteness predicted perceived metaphoricity. This set of norms, the first of its kind, serves as a resource for research employing a variety of computational, behavioral, and neuroimaging methods to examine the nature of metaphor comprehension and semantic composition.

## Introduction

Metaphorical expressions such as *My lawyer is a shark* or *Mary is an early bird* jazz up our language use. What makes them particularly appealing to empirical investigation is that the contents they convey ultimately differ from what the expressions mean literally. Given that the interpretation of metaphorical expressions appeals to cognitive resources that go beyond linguistic denotation, their investigation in isolation and utterance contexts constitutes a window into how the brain composes meaning and how language interfaces with other cognitive systems. Research on metaphors is thus at the confluence of a variety of issues bearing on the nature of human cognitive architecture.

In this research context, metaphorical norms serve two main goals. The first goal is to enable experimental studies to rely on more robust sets of materials than those devised within a specific laboratory or designed for a specific experiment conducted with a relatively small set of materials and sample size. Norming studies are important methodologically because they allow laboratories and research groups to investigate different hypotheses about the processing, representation, and neuronal implementation of metaphors while relying on the same source of materials, thus facilitating cross-experimental comparisons. In addition, norms allow for better replication of experiments—which is key to the validity of purported psychological and neurological mechanisms—by contributing to the standardization of materials (Nosek et al., 2015). Besides serving as an open source of experimental materials, a study on norms further contributes to understanding how metaphorical expressions are interpreted and how their processing is implemented in the brain. Regarding this second goal, a norming study can also be considered an “offline” (that is, not in real time) processing experiment, one in which properties of expressions can be tested for different factors and under different methods and interpretive conditions.

The present study has these two main goals. First, we provide norms for 300 two-word metaphor combinations—such as *broken heart* and *early bird*—presented in sentence context and in isolation. These norms include (a) the linguistic structure of the expressions, (b) frequency, and ratings of (c) familiarity, (d) aptness, (e) concreteness, (f) metaphoricity, and (g) metaphor constituent structure. Second, these two presentation formats also enabled us to examine the effect of context on our main variables of interest—familiarity and aptness—which are well known to play a key role in metaphor comprehension (Blasko & Connine, 1993).

Familiarity is an important variable in metaphor research because it reflects the extent to which participants have heard or read the expression in the past. The assumption is that the more familiar the expression is, the easier it is to recover its content from memory or to compose its meaning during comprehension (Gentner & Bowdle, 2008).

Aptness reflects the extent to which one word captures important properties of another in order to convey figurative content. In copular metaphors, such as *My lawyer is a shark*, the vehicle *shark* is used to predicate some property of *lawyer*—perhaps that of being ruthless, sneaky, or aggressive. In the two-word expressions employed in the present study, such as in *broken heart*, the relation between “topic” and “vehicle” is less evident. In the case of *broken heart*, *broken* may be used to convey the rupture of an emotional state conveyed by *heart* (usually referring to love). Therefore, in *broken heart* the vehicle *broken* is predicating some state of rupture of the heart—or some love gone awry. Aptness, in the present context, then, means how appropriate it is to predicate this sense of rupture of an emotional state such as love. As several studies have shown, aptness plays a central role in the process of attaining the metaphorical content of a given expression (Chiappe & Kennedy, 1999; Chiappe et al., 2003; Roncero & de Almeida, 2014)—a role that is often taken to be greater than familiarity (Roncero & de Almeida, 2014). Thus, while familiarity reflects the usage properties of a given expression, aptness reflects the degree of semantic appropriateness of an expression, independent of how familiar it may be.

In the present study, familiarity and aptness were investigated both as baseline conditions (in isolation) and when the expressions were embedded in metaphorical contexts. Given that metaphor interpretations are largely contextually driven, the same word combinations can be interpreted literally or metaphorically depending on the utterance context. For instance, *cold feet* could refer to either actual feet being cold due to the weather or to an emotional state such as a lack of confidence. Thus, deviations in sensitivity to familiarity or aptness can be taken as the effect of context on an expression’s interpretation.

Concreteness is an important factor in metaphor comprehension, as it can ease interpretation, particularly of less familiar metaphors. In the present norms, we assessed the concreteness of the concept conveyed by the metaphorical vehicle itself, rather than word-level concreteness available in other sources (e.g., Brysbaert et al., 2014). For example, a concrete vehicle like *moustache* in *milk moustache* is relatively easy to interpret, as it represents the visible shape of a milk stain that can be visually perceived or imagined. In contrast, an abstract vehicle, such as *soft* in *soft skills,* can be more challenging to comprehend, as it denotes intangible qualities, irrespective of the concreteness of the word *soft* itself.

Metaphoricity has been identified as one of the “big two” dimensions influencing metaphor comprehension (Thibodeau et al., 2018) and refers to the extent to which an expression is perceived as figurative rather than literal. This dimension is particularly important because some two-word expressions (e.g., *early bird*) originate from idiomatic phrases (e.g., *the early bird catches the worm*). Such expressions can become lexicalized and may no longer be perceived as metaphorical. Expressions without idiomatic origins can also vary in figurativeness; for example, *fan brush* may be interpreted as highly metaphorical (a makeup brush shaped like a fan) or as more literal (a brush used as a fan).

Thus far, there have been relatively few large metaphor norming studies, with most employing the more traditional and productive copular form (*x is y*), with their differences being only on the nature of the *x* and *y* constituents. The majority of Katz et al.’s (1988) items, for instance, include long topic and vehicle phrases (e.g., *The creative mind is a kettle on the stove*, *Thunderclouds are wild horses galloping across the sky*; see also Campbell & Raney, 2015). In Roncero and de Almeida’s (2015) norms, all expressions had simple noun phrases in the topic and vehicle positions for both metaphors and simile forms (e.g., *Roads are snakes*/*Roads are like snakes*). Other studies (Cardillo et al., 2010, 2017) have provided norms for different metaphorical constructions, such as verb phases (e.g., *The insults hopped on her tongue*), but also complex copular ones (e.g., *The editorial was a brass-knuckle punch*) together with literal sentences of the same form.

What is unique about the present metaphor norms is that they are the first to be obtained for two-word metaphorical expressions, such as *broken heart*, *early bird*, and *sharp mind* in isolation and in sentence context. Although the relationship between the two words in these expressions is, on the surface, relatively simple, on closer inspection, they carry many syntactic and semantic intricacies. To begin with, these expressions include a variety of syntactic categories, but predominantly adjective-noun (Adj-N), such as *broken heart* and noun-noun (N–N), such as *drug mule*. These expressions are also complex in how the two words are used metaphorically. We will use the term “vehicle” to refer to the word that carries most of the metaphorical content (e.g., *sharp*) and “topic” to refer to the constituent that is predicated on (e.g., *mind*). It can also be the case that both constituents work together as the vehicle to describe a topic that remains implicit, as it is the case of *smoking gun* used to refer to a type of evidence that is incontrovertible, not to a type of gun. In most cases, however, the vehicle is the first word of the pair (e.g., *dark*), and it is followed by the topic, which can maintain its literal meaning (e.g., *personality*) or bear another figure of speech, such as metonymy (e.g., *mind*). Thus, contrary to the topic and vehicle of copular metaphors, in which the topic (e.g., *lawyer*) precedes the word used for predication (e.g., *shark*), in most two-word metaphors, the vehicle precedes the topic. In this way, these expressions behave as English compound words, whose modifier is the first constituent, and the head is the second (e.g., *heartbreak*).^1^ This is typically the case with very productive adjectives, such as *bright,* which is conventionally used to refer to someone or something intelligent (*bright student*, *bright idea*, etc.). In other cases, only the second word, the vehicle, is metaphorical. For instance, in *early bird*, *bird* is used to refer to a person who does things in the morning or sooner rather than later. However, the relation between the two words is not always clear. For instance, *sharp mind* predicates “sharpness” of mind, but *mind* is used metonymically to refer to someone’s intellectual ability, with only *sharp* being used metaphorically to express the idea of someone being able to think fast and clearly. In the present norms, the distinction between metaphorical and metonymic uses of a word is often blurred given that both are considered figurative forms (i.e., their meanings do not come from the object that they refer to—such as in *mind*—but from how the word is used to refer or predicate of something or someone). To capture these nuances, we collected metaphor constituency ratings, which indicate whether the metaphorical content is carried primarily by the first word, the second word, or is distributed across both.

A third way in which the expressions we studied vary is whether the expression has an equivalent literal use—such as *cold feet*—or if it can only be interpreted figuratively, as in *broken heart*. The advantage of embedding these expressions in context is, thus, that we can ensure that the ratings provided in a biasing context refer to the metaphorical content, rather than to the literal meaning. Further, context has been shown to play a role in the way metaphors are processed. For instance, Bambini et al. (2016) examined the role of context during metaphor comprehension by contrasting literal and metaphorical expressions in both minimal and supportive contexts (i.e., where the ground—the relationship between the topic and vehicle—of the metaphor was made explicit). They found that, when expressions were presented in minimal context, metaphors yielded greater N400 and P600 amplitudes in comparison to their literal counterparts. In contrast, when expressions were presented in a supportive context, metaphors showed a reduced N400 amplitude, thus matching their literal counterparts.

Before we present our norms, it is important to note that, to our knowledge, there have been few psycholinguistic studies investigating the nature and processing of two-word metaphorical expressions (Al-Azary et al., 2021; Arzouan et al., 2007; Briner et al., 2018; Forgács et al., 2015; Gagné, 2002; Goldstein et al., 2012; Kavé et al. 2014; Park, 2021; Pissani & de Almeida, 2022, 2023). For instance, Gagné (2002) studied exclusively noun-noun combinations (e.g., *closet heart, trumpet voice*) adapted from copular metaphors (e.g., *Hearts are closets, some voices are trumpets)*, and found that factors influencing the comprehension of copular metaphors (i.e., aptness, salience, and expectancy) also affected two-word metaphor combinations. Similarly, Forgács et al. (2015) examined novel adjective-noun combinations where the same head was preceded by an abstract modifier (e.g., *conditional schedule*), a concrete modifier (e.g., *printed schedule*), or a metaphorical modifier (e.g., *thin schedule*). Their results suggest that the modifier affects how the whole expression is processed, such that reading the same noun may yield a larger concreteness effect following a concrete adjective rather than an abstract one. Interestingly, metaphorical word pairs rated more abstract yielded a larger N400 effect on the noun, when compared to the noun of word pairs rated more concrete.

More recently, Al-Azary et al. (2021) examined modifier-noun phrases (e.g., *shark lawyer*) and found that—similar to copular metaphors (e.g., *My lawyer is a shark*)—these expressions were subject to the metaphor interference effect (Glucksberg et al., 1982), in which the metaphorical content is taken to be processed automatically, even when a literal interpretation would suffice. It is important, however, to highlight the heterogeneity of the materials employed in these studies, with most being adapted and rated ad hoc.

It is also important to note that investigating these metaphorical expressions plays a key role in understanding compositionality. Crucially, compositionality bears on how an expression’s meaning is a function of its constituents and how they are structured—for example, whether *cold feet* is a function of the meaning of *cold* and *feet* and their predication relation, such that *cold* is predicated on *feet*. Compositionality is one of the fundamental characteristics of human cognitive architecture and it is said to underly the productivity of our linguistic and conceptual systems (de Almeida & Lepore, 2018; Fodor & Lepore, 2001; Fodor & Pylyshyn, 1988; Partee, 1995). But clearly, not all linguistic expressions appear to compose the same way: while *cold feet* may be compositional on its own when it is inserted in a metaphorical context, its sense differs from the meanings of its constituents and how they combine. Compositionality of linguistic expressions is then crucially linked to the literal/metaphorical distinction (Gibbs & Colston, 2012). Two recent studies (Pissani & de Almeida, 2022, 2023) have shown that even in sentences such as *Most people agree that a broken heart can be difficult to overcome,* where the metaphorical interpretation is biased*,* the literal meaning of the expression remains active for several seconds after the expression has been processed, suggesting that literal interpretation—and, thus, composition—is attained even in metaphorical contexts. That is, these expressions require a local composition besides the content that the two-word expression contributes to its carrier sentence.

### The present norms

We collected norms for 300 metaphorical expressions in isolation and in sentence contexts. All expressions in our set are of the form *YX*, with words representing two grammatical combinations, adjective + noun (*N* = 187) and noun + noun (*N* = 113). Of these, 147 expressions carry their metaphorical content in the first word (e.g., *bright student*), 63 expressions in the second word (e.g., *old flame*), and 90 expressions in both words (e.g., *smoking gun)*. Another dimension of our set is that some of these expressions are always interpreted metaphorically (e.g., *emotional rollercoaster*), while others may also be interpreted literally (e.g., *cold turkey*). We also included COCA frequency scores for the co-occurrence of both words that constitute the combination, as well as scores for the individual constituents. For this set of expressions, we have examined the relationships among the variables by computing Pearson’s correlation coefficients for familiarity, aptness, concreteness, metaphoricity, constituency, and frequency. Lastly, we present analyses of the effects of context on familiarity and aptness ratings; of vehicle position on aptness, concreteness, and metaphoricity; and of the combined influence of familiarity, concreteness, and vehicle position on metaphoricity. The full set of norms is available at https://osf.io/xk3j9.

## Method

### Participants

A total of 164 native English speakers, aged 19 to 70 years (M = 40.3, SD = 11.9; 97 male), participated in this study. Of these, 54 participants completed the familiarity rating task, 49 participants completed the aptness rating task, 26 participants completed the concreteness rating task, 25 participants completed the metaphoricity rating task, and ten participants completed the metaphor constituency task. All participants reported having normal or corrected-to-normal vision and had no history of reading or hearing disabilities.

Data from 34 additional participants were excluded before data analyses as detailed in the Data preparation section. Participants were recruited via Amazon Mechanical Turk (MTurk) and were restricted to those residing in countries where English is the official or dominant language (e.g., Canada, the US, the UK). Only MTurk workers with an approval rating of 80% or higher were eligible. Each participant received CAD$12 for their participation.

### Materials

We compiled a total of 300 two-word metaphor combinations. Of these expressions, 250 were sourced from everyday conversations and diverse media outlets such as newspapers, blog posts, streaming services, video-sharing platforms, and social media. The remaining 50 expressions were selected from Briner et al. (2018), which provided local norms for 100 two-word combinations categorized as 25 conventional metaphors, 25 novel metaphors,^2^ 25 literal pairs, and 25 unrelated pairs. From their dataset, we included the 25 conventional metaphors and 25 novel metaphors. All expressions were embedded in sentence contexts created by research assistants who are native English speakers and further curated by the authors. These contexts were designed to support metaphorical interpretations and provide a consistent frame of reference for participants, though we did not manipulate context type as in previous work (e.g., Thibodeau et al., 2018). For all expressions, we compiled a range of linguistic variables and collected subjective ratings of familiarity and aptness (both in context and in isolation), concreteness, metaphoricity, and metaphor constituency. These variables can be further defined as follows (see Table 1 for a summary).

**Table 1:** Summary of all the variables present in the norming study

Variable	Abbreviation	Description
Syntactic structure	structure	describes the grammatical structure of the metaphorical combination, whether Adj-Noun (AN) or Noun-Noun (NN)
Topic	topic	names the subject of the metaphor being described by the expression
Vehicle	vehicle	names the word used to describe the topic of the metaphorical expression
Vehicle position	vehicle_position	indicates whether the metaphorical content is carried in the first, second, or both words of the expression
Metaphor constituency	CONS	provides a graded measure of the perceived position of the vehicle within an expression
Literalness	literalness	marks whether the expression can be interpreted literally (1) or only metaphorically (0)
Context	context	The sentence in which the expression appeared when presented to participants in context during the rating tasks
COCA Frequency Score	FREQ	provides the COCA frequency score for the co-occurrence of both constituents of the expression
	FREQ _modifier FREQ_head	provides the COCA frequency score for either the first (modifier) or second (head) of the expression
Familiarity	FAMc FAMi	provides subjective familiarity ratings separately for expressions embedded in context (c) or presented in isolation (i)
Aptness	APTc APTi	provides subjective aptness ratings separately for expressions embedded in context (c) or presented in isolation (i)
Concreteness	CON	provides subjective concreteness ratings for the conveyed meaning of the vehicle
Metaphoricity	MET	provides subjective metaphoricity ratings for expressions embedded in context

#### Syntactic structure

All expressions are composed of two words, where the first word (modifier) is either an adjective (e.g., *red flag*) or a noun (e.g., *drug mule*), while the second word (head) is always a noun.

#### Metaphor constituency

The metaphorical content (vehicle) of an expression can be carried by the modifier, the head, or both. For instance, in expressions such as *bright student*, the modifier carries the metaphorical content and can be paired with different nouns (*bright student*, *bright author*, *bright idea*, etc.). In this case, all instances of *bright* play the same role, that is, predicating some form of “cleverness” to the head noun referent. For those cases, we selected only one occurrence (*bright student*). However, we included expressions with the same modifier only if they contributed unique semantic content to the expression. For instance, in *bright side*, *bright* is used as “hopeful” or “promising”, while in *bright student, bright* is used as “intelligent” or “clever”. Conversely, some expressions carry the metaphorical content in the head, but the modifier can be interpreted literally. For instance, in expressions such as *old flame*, the modifier *old* can be literally interpreted as “former”, while the head *flame* can be figuratively interpreted as “lover”. In some cases, metaphorical heads can be paired with different modifiers as is the case of *old flame* and *new flame* or *red flag* and *green flag,* in which cases, we selected the most frequently used. Finally, other expressions carry the metaphorical content in both words. For instance, the expression *smoking gun* is used together to refer to conclusive evidence. In our norms, *vehicle position* indicates whether the metaphor content is carried by the first word (modifier), the second word (head), or both words, coded categorically based on the authors’ judgement. In addition, we provide *metaphor constituency* as an empirical, graded measure capturing native speakers’ intuitions about how the metaphorical content is distributed across both words.

#### Literalness

Most of the expressions can be understood only metaphorically (e.g., *emotional rollercoaster*). However, some can be interpreted both metaphorically and literally. In some of these expressions, the metaphorical meaning may be more salient (e.g., *cold turkey*), while in others the literal meaning may be more salient (e.g., *hot water*). It is important to note that participants were aware that they were rating metaphorical expressions. Thus, even in the case of metaphors that can also be understood literally, ratings are meant to reflect their metaphorical interpretation.

#### COCA frequency score

We included frequency scores obtained from the Corpus of Contemporary American English (COCA; Davies, 2008) for each word separately, as well as for the co-occurrence of both words that compose the two-word combination. COCA is a large, long-established English corpus and contains over one billion words. It is important to mention that the COCA database does not distinguish between metaphorical and literal uses, thus expressions like *hot water* (5124) and *big brother* (3766) had the highest scores, while the most frequent exclusively metaphorical expression was *movie star* (2687). Expressions with the lowest frequency scores included *penguin huddle* (1) and *burning moment* (1), while 31 expressions did not appear in the database and were marked with a frequency score of zero. Among the latter, some were contemporary expressions (e.g., *online scrub*), domain-specific (e.g., *spider plank*), and others were created or adapted by previous researchers (e.g., *gentle art*).

### Procedure

The studies were programmed using Psychopy3 (Versions 2021.2.3 and 2024.2.4; Peirce et al., 2022), with stimulus presentation and data collection conducted via the Pavlovia online platform. Each participant completed only one of the following tasks: (a) the familiarity rating task, (b) the aptness rating task, (c) the concreteness rating task, (d) the metaphoricity rating task, or (e) the constituency ratings task. After receiving a link to our study, participants were first directed to a virtual consent form, followed by a demographic questionnaire. For the tasks presented in context, participants viewed a sentence containing the metaphorical expression in upper case (e.g., *The EARLY BIRD always gets the best seat at the movie theatre*). For the tasks presented in isolation, only the metaphorical expression was shown (e.g., *EARLY BIRD*). After a 3-s delay, a 1-to-7 numerical scale appeared below the sentence or expression. Participants were instructed to rate the expression in uppercase, with specific instructions depending on the assigned task. To ensure attention, additional trials were included as attention checks. These trials required participants to press a specific number on the scale (e.g., *For this sentence, press the number five so we know you are paying attention.*), and appeared once per block at random.

#### Familiarity rating collection

Participants were asked to rate metaphorical expressions for familiarity, both in context and in isolation. Participants were encouraged to use the full scale, with not-so-well-known expressions rated more towards the middle (2, 3, 4, 5, 6), reserving 1 for truly not familiar expressions and 7 for very familiar ones. In the instructions, familiarity was defined as the extent to which participants had heard or read each expression in the past. Two detailed examples, including a familiar and a less familiar expression, were provided. For instance, if the expression ***feeling blue*** was a well-known expression to participants, they were advised to give it a high rating (perhaps 6 or 7), whereas if ***crying wolf*** was not as well-known, they were advised to provide a lower rating (perhaps 2 or 3). Familiarity ratings were collected in two blocks with context as a within-subjects factor, which was counterbalanced in lists one and two. In list one, participants rated half of the expressions in context and the other half in isolation, while the opposite was true for list two. Participants were assigned to only one list and encouraged to take a break between blocks.

#### Aptness rating collection

Participants were asked to rate metaphorical expressions for aptness, also in context and in isolation. In the instructions, aptness was defined as the extent to which the vehicle captures important features of the topic. Six examples, including apt and inapt expressions with different vehicle positions, were provided. For instance, participants were instructed that the expression *silky hair* could be considered a highly apt expression because *silky* captures important features of the hair (namely, shininess and smoothness). On the other hand, *silky sunset* may be considered a less apt expression since it is less common for sunsets to be both shiny and smooth. Therefore, *silky hair* would receive a high rating (perhaps 6 or 7), whereas *silky sunset* would receive a lower rating (perhaps 3 or 4).

Aptness ratings were collected in three blocks with the vehicle position as a within-subjects factor and context as a between-subjects factor, both of which were counterbalanced in lists three and four. In both lists, expressions were divided into three blocks (A, B, and C) according to the position of the vehicle to facilitate the rating task. Thus, block A contained expressions where only the modifier was metaphorical (e.g., *bright student*), block B contained expressions where only the head was metaphorical (e.g., *early bird*), while block C contained expressions where both words were metaphorical (e.g., *red flag*). In list three, all expressions were shown in context, and blocks appeared in consecutive order (i.e., A, B, C). In list four, all expressions were presented in isolation, and the order of the blocks differed from list three (i.e., B, C, A).

#### Concreteness rating collection

Participants were asked to rate metaphorical expressions for concreteness. In the instructions, concreteness was defined as the extent to which the conveyed meaning of the vehicle could be perceived through the senses (smelling, tasting, touching, hearing, seeing) or through actions (e.g., pointing at an object or showing a picture of it). Four examples, including both concrete and abstract meanings with different vehicle positions, were provided to participants. For instance, participants were told that in the expression *zebra crossing*, the vehicle *zebra* conveys “marked with stripes,” which is easily perceived visually. Consequently, *zebra crossing* would receive a higher rating (perhaps 6 or 7). Conversely, in the expression *truth bomb*, the vehicle *bomb* conveys “blunt or unexpected,” which is not easily perceived by the senses or through actions. Therefore, *truth bomb* would receive a lower rating (perhaps 2 or 3). For each expression, the vehicle was written in uppercase, and the topic was written in lowercase, with an example of the expression embedded in a sentence presented underneath. Concreteness ratings were collected in three blocks with the vehicle position as a within-subjects factor in list five.

#### Metaphoricity rating collection

Participants were asked to rate expressions for metaphoricity. In the instructions, metaphoricity was defined as the extent to which the expression was considered literally or metaphorically true within the context of the sentence. Three examples, representing low, medium, and high levels of metaphoricity, were provided. Participants were instructed that, for the low level, *ROCKY TRAIL* (*The hikers walked carefully along the rocky trail through the forest*) describes a physical path covered with rocks. This is a literal description of terrain and would therefore receive a low rating, perhaps 1 or 2. For the medium level, *SOFT WARNING* (*The teacher gave the students a soft warning before starting the exam*) conveys a mild or gentle warning. Because a warning cannot literally be soft or hard, this expression is somewhat metaphorical and would receive a mid-level rating, perhaps 3 or 4. For the high level, *SMOKE SCREENS* (*The politician’s promises were just smoke screens to distract the public from real issues*) refers to something intended to obscure or mislead, rather than literal smoke or screens, and would receive a high rating, perhaps 6 or 7. Metaphoricity ratings were collected in two blocks of 150 sentences each, allowing participants to take a break in between.

#### Constituency rating collection

Participants were asked to rate how the metaphorical content was distributed across the two words of the expressions. Constituency reflects the extent to which the metaphorical content is carried by the first word, the second word, or both words, using a graded measure (see Table 2). It is, thus, a form of measure of metaphor meaning composition. Three examples were provided for participants to illustrate each case. They were instructed that, in *SNAIL MAIL,* the metaphorical content is carried by the first word *snail* (suggesting slowness), while *mail* remains literal, which would receive a low rating towards – 3. In *TIME BOMB*, both words contribute to the metaphorical content, with *time* adding urgency and *bomb* adding danger, which would receive a middle rating towards 0. In *MENTAL PRISON*, the metaphorical content is carried by the second word *prison* (suggesting confinement), while *mental* remains literal, which would receive a high rating towards 3. Observed constituency scores aligned with the authors’ classification of the vehicle position. Expressions classified as having the vehicle in the first word (*N* = 147) had a mean constituency rating of – 2.09 (*SD* = 0.70); those classified as having the vehicle in both words (*N* = 90) had a mean of – 0.17 (*SD* = 0.88); and those whose second word carried the metaphorical content (*N* = 63) had a mean of 1.47 (*SD* = 1.04).

**Table 2:** Rating scale used for metaphor constituency

Rating	Definition
– 3	*Definitely in the first word*
– 2	*Mostly in the first word*
– 1	*Slightly more in the first word*
0	*Equally in both words*
1	*Slightly more in the second word*
2	*Mostly in the second word*
3	*Definitely in the second word*

### Data preparation

For our analyses, we only included data from eligible participants who correctly followed the instructions. Thus, we removed 11 participants for not using the full rating scale (e.g., using 5 to rate all expressions), nine participants for incorrectly responding to at least one attention check, two participants for having previously participated in a different version of the study, and 12 participants for not finishing the task.

### Data analysis

We conducted cumulative link mixed-effects regression analyses on our ordinal data using the clmm function from the *ordinal* package in R (Christensen, 2018; R Core Team, 2020). Model comparisons were conducted using likelihood ratio tests, in which the fit of the final model was evaluated against that of reduced models.

The response variables—familiarity, aptness, concreteness, and metaphoricity ratings—were measured on an ordinal scale from 1 to 7. The predictor variable *context* was treated as a binary categorical variable with levels for presence and absence. The predictor variable *vehicle position*, which indicates the word carrying the metaphorical content, was a categorical variable with levels for the first word, the second word, and both words. For the metaphoricity model, we included *familiarity, aptness*, and *concreteness* as predictors to examine which factors contribute to some expressions being perceived as more metaphorical than others.

In the familiarity rating task, *context* was manipulated within participants across blocks (i.e., participants rated expressions in context in one block and in isolation in the other) and it was included as a fixed effect in the familiarity model. In the aptness rating task, context was manipulated between participants, while vehicle position was manipulated within participants across blocks; thus, context, vehicle position, and their interaction were included as fixed effects in the aptness model. In the concreteness rating task, vehicle position was manipulated within participants across blocks and was included as a fixed effect, while context was not manipulated and therefore not included. In the metaphoricity rating task, vehicle position was manipulated within participants across blocks and was included as a fixed effect, along with familiarity, aptness, and concreteness; context was not manipulated and therefore not included. Across all models, random intercepts for participants and items, as well as random slopes for the effect of context within items, were included.

## Results

### Descriptive statistics and correlations for familiarity, aptness, concreteness, metaphoricity, and constituency.

Table 3 presents descriptive statistics and correlations for familiarity in context (FAMc), familiarity in isolation (FAMi), aptness in context (APTc), aptness in isolation (APTi), concreteness (CON), metaphoricity (MET), metaphor constituency (CONS), and frequency scores for the expression obtained from COCA (FREQ). Pearson correlations were computed between all variables, and the associated p-values were adjusted for multiple comparisons using the Holm-Bonferroni method. Correlations were considered statistically significantat *p* <.01.

**Table 3:** Descriptive statistics and correlations for all variables included in the norming study

	Descriptive statistics			Correlations						
	M	SD	Range	FAMc	FAMi	APTc	APTi	CON	MET	CONS
FAMc	4.93	0.84	2.82–6.21	-						
FAMi	4.83	0.93	2.57⁠⁠–6.42	.87*	-					
APTc	4.74	0.69	2.88⁠–6.12	.75*	.67*	-				
APTi	4.47	0.61	3.04⁠⁠–6.38	.71*	.76*	.73*	-			
CON	3.58	0.91	⁠⁠1.96–6.46	–.16	–.21*	⁠⁠.01	–.16	-		
MET	4.83	1.16	1.48–6.68	.06	.11	–.27*	.00	–.40*	-	
CONS	– 0.77	1.65	– 3.00 to 3.00	.13	.23*	–.10	.10	–.08	.39*	-
FREQ	324	574	0–5124	.43*	.44*	.28*	.30*	–.20	0.10	.09

Asterisks (*) mark statistically significant correlations at *p* <.01

As expected, strong positive correlations were observed between familiarity in isolation (FAMi) and familiarity in context (FAMc), as well as between aptness in context (APTc) and aptness in isolation (APTi). All correlations between familiarity and aptness ratings were also strongly positive. Concreteness (CON) showed a weak negative correlation with familiarity in isolation. Metaphoricity (MET) was negatively correlated with aptness in context and concreteness, the latter indicating that more concrete expressions tend to be perceived as less metaphorical. Metaphor constituency (CONS) was positively correlated with metaphoricity, suggesting that expressions in which the metaphorical content is carried by the second word tend to be perceived as more metaphorical. Frequency scores (FREQ) were moderately positively correlated with familiarity and aptness, both in context and in isolation.

### Regression models for familiarity, aptness, concreteness, and metaphoricity

#### Familiarity

We examined the effect of context on familiarity ratings. The full model, including the effect of context, was a significantly better fit to the data than the null model, χ^2^(3) = 20.04, *p* <.001. Context had a significant effect, indicating that the presence of context increased familiarity ratings (logit coefficient = 0.08, SE = 0.03, z = 2.61, *p* <.01). We then used the *emmeans* package in R (Lenth, 2024) to calculate the predicted probabilities of observing higher ratings in each condition. Probabilities were 0.72 for expressions presented in isolation, increasing to 0.74 when context was present. Overall, participants were very likely to give higher ratings for all expressions, with the likelihood increasing minimally by approximately 2% when context was present.^3^

#### Aptness

We examined the effects of context and vehicle position on aptness ratings. The full model, including the effect of context and of vehicle position, was a significantly better fit to the data than the null model, χ^2^(5) = 31.34, *p* <.001. In contrast to familiarity, context was not a significant predictor of aptness rating (logit coefficient = 0.32, SE = 0.22, *z* = 1.40, *p* =.16). Vehicle position, however, did affect ratings, with lower aptness ratings when the vehicle was the first word (logit coefficient = – 0.11, SE = 0.05, *z* = – 2.00, *p* =.05). Estimated probabilities of observing higher aptness ratings were 0.64 when the vehicle was the first word, 0.67 when it was the second word, and 0.68 when it was both words.

#### Concreteness

The effect of vehicle position on concreteness ratings was also examined. The full model, including the effect of vehicle position, was a significantly better fit to the data than the null model, χ^2^(2) = 57.85, *p* <.001. Vehicle position influenced ratings, with higher concreteness ratings when the vehicle was the first word (logit coefficient = 0.94, SE =.13, *z* = 7.28, *p* <.001) or the second word (logit coefficient = 1.02, SE =.16, *z* = 6.47, *p* <.001) compared to both words. Estimated probabilities of observing higher concreteness ratings were 0.45 when the vehicle was the first word, 0.47 when it was the second word, and 0.24 when it was both words.

#### Metaphoricity

We also examined whether familiarity, aptness, concreteness, and vehicle position influence perceived metaphoricity. The full model, including all four predictors, was a significantly better fit to the data than the null model, χ^2^(5) = 194.32, *p* <.001. More familiar expressions (logit coefficient = 0.42, SE =.13, *z* = 3.15, *p* =.002) were rated as more metaphorical. In contrast, more apt (logit coefficient = – 0.90, SE =.16, *z* = – 5.68, *p* <.001) and more concrete expressions (logit coefficient = – 0.40, SE =.08, *z* = – 4.85, *p* <.001) were rated as less metaphorical. Vehicle position also affected ratings, with higher metaphoricity ratings when the vehicle was the second word (logit coefficient = 1.20, SE =.19, *z* = 6.44, *p* <.001) or both words (logit coefficient = 1.57, SE =.18, *z* = 8.90, *p* <.001) compared to the first word. Estimated probabilities of observing higher metaphoricity ratings were 0.60 when the vehicle was the first word, 0.83 when it was the second word, and 0.88 when it was both words.

#### Reliability

The reliability of subjective ratings for familiarity, aptness, concreteness, metaphoricity, and constituency was assessed using the intraclass correlation coefficient (ICC), calculated with the *psych* package in R (Revelle, 2025). ICC values were based on mean ratings, consistency, and two-way mixed-effects models. For familiarity, reliability scores are reported for the subset of expressions rated by the same sample, as context was measured between participants. The results of this analysis are summarized in Table 4. Following the guidelines of Koo and Li (2016, p.161), ICC values indicated moderate reliability for APTi, good reliability for FAMc, FAMi, APTc, and CONS, and excellent reliability for MET and CONS.

**Table 4:** Intraclass correlation coefficient (ICC) values for subjective ratings of familiarity, aptness, concreteness, metaphoricity, and constituency

	ICC	95% Confidence interval	Number of items	Number of raters
FAMc	0.82	[0.77, 0.86]	150	26
	0.88	[0.85, 0.91]	150	28
FAMi	0.87	[0.84, 0.90]	150	26
	0.88	[0.85, 0.90]	150	28
APTc	0.78	[0.74, 0.82]	300	25
APTi	0.70	[0.65, 0.75]	300	24
CON	0.87	[0.84, 0.89]	300	26
MET	0.94	[0.93, 0.95]	300	25
CONS	0.93	[0.91, 0.94]	300	10

These findings suggest that participants’ subjective ratings were consistent, supporting the use of familiarity, aptness, concreteness, metaphoricity, and constituency as meaningful constructs. The high internal consistency provides confidence in the interpretation of the relationships among these variables within the context of the study, strengthening the validity of our findings.

These results are discussed next, considering their potential contribution to future studies and what they inform us about the nature of two-word metaphor comprehension.

## Discussion

We collected norms for 300 metaphorical two-word combinations such as *broken heart* presented in context (e.g., *She was left with a broken heart after the split with her partner*) and in isolation.

We had two main goals with the present norms. First, we sought to provide a robust set of experimental materials to support the execution of experiments investigating the representation, processing, and neuronal implementation of metaphors. As an open source, our norms promote the standardization of materials and allow for comparison across studies. Conversely, local norms that are collected ad hoc may be limited to a smaller set of materials, sample size, and time frame. Second, we aimed to contribute to the understanding of metaphor interpretation and implementation in the brain by treating our norms as offline data. It is important to note that several metaphor processing studies have also relied on “offline” methods to test the nature of metaphor comprehension. Among these offline methods are semantic judgement (McElree & Nordlie, 1999), sentence verification (Glucksberg et al., 1982), completion tasks (Bowdle & Gentner, 2005), internet usage data (Roncero et al., 2016), multiple-choice tasks (Stamenković et al., 2019), and sensory modality ratings (Winter & Strik-Lievers, 2023). In the same vein, our norms provide data obtained offline—for instance, the contrast between familiarity ratings in context versus in isolation—to inform about the nature of metaphor interpretation. While this was not our main goal, we argue that contextual information is crucial for understanding metaphorical expressions of the kind we used here.

We collected ratings for familiarity, aptness, concreteness, metaphoricity, and metaphor constituency, which are integral factors in metaphor comprehension (Katz, 1989; Blasko & Connine, 1993; Chiappe & Kennedy, 1999; Chiappe et al., 2003; Bowdle & Gentner, 2005; Jones & Estes, 2006; Roncero & de Almeida, 2014; Roncero et al., 2016; Al-Azary & Buchanan, 2017; Thibodeau et al., 2018). Familiarity and aptness ratings were collected both in context and in isolation, concreteness and metaphoricity ratings were collected with support from context, and metaphor constituency was collected in isolation. Context has also been shown to modulate metaphor interpretation (Bambini et al., 2016; Janus & Bever, 1985). In our study, we first examined the effect of context on both familiarity and aptness. Expressions were perceived as more familiar, but not more apt, when presented in context than when presented in isolation. These findings are partially compatible with the idea that subjective ratings of familiarity and aptness are confounded with processing fluency (Thibodeau & Durgin, 2011; Thibodeau et al., 2018). That is, participants may base their ratings on how easily they understand the sentences. Thus, the biasing context in which our expressions were embedded may have facilitated their comprehension, which in turn yielded higher familiarity ratings. Aptness ratings, however, did not vary with context, which reinforces the view that the two—aptness and familiarity—are different constructs. Familiarity reflects how often an expression is encountered—and can be therefore influenced by context—whereas aptness reflects the perceived quality of the expression, independent of context.

Vehicle position is another important variable in two-word metaphor combinations. Unlike copular metaphors (e.g., *My lawyer is a shark*), where the topic precedes the vehicle, two-word metaphors show much more variation. In these expressions, the metaphorical content may be carried by the first word, the second word, or distributed across both words. For instance, Pissani and de Almeida (2023) found that the literal meaning was available further during sentence comprehension only for expressions in which the first or second word was metaphorical, but not both. This suggests that, after the metaphorical content has been attained, the individual concepts decay, which is expedited when both constituents of the expression are metaphorical. The present study also shows that vehicle position influences ratings on other metaphorical dimensions. When the first word carries most of the metaphorical content (e.g., *bright student*), expressions are perceived as less apt but more concrete. When the second word carries most of the metaphorical content (e.g., *social butterfly*), expressions are perceived as more concrete and more metaphorical. When the metaphorical content is distributed across both words (e.g., *silver lining*), expressions are perceived as less concrete but more metaphorical. This pattern may be attributable to the nature of the expressions in our dataset: single-word vehicles often describe the shape of a tangible object (e.g., *noodle, moustache, almond, hourglass, tent*), which is visually perceptible. In contrast, vehicles composed of two words tend to denote more abstract concepts (e.g., *sixth sense, silver lining, cloud nine*), which are less directly visualizable. Although our regression model shows a negative relationship between metaphoricity and concreteness, expressions appear more metaphorical when the metaphorical content shifts to the second word or is distributed across both words, as reflected by the negative correlation between metaphoricity and constituency, independent of concreteness. Note that this may also be an artifact of our dataset, as the number of expressions in each vehicle position category is uneven.

We also examined whether familiarity, aptness, concreteness, and vehicle position contributed to the perceived metaphoricity of the expressions. Metaphors that were more familiar were perceived as more metaphorical (e.g., *smoking gun, broken heart*), suggesting that participants recognize figurative content more easily when an expression is familiar. This effect may seem counterintuitive for conventional expressions and frozen metaphors (e.g., *rough day, restaurant chain*), which over time become lexicalized and lose their association with the original content (Bowdle & Gentner, 2005; Keysar et al., 2000). Conversely, metaphors that were more apt were perceived as less metaphorical (e.g., *weak argument, clear intention*), suggesting that expressions that are highly appropriate, and therefore used frequently, may no longer be recognized as metaphorical. Metaphors with more concrete topics were also perceived as less metaphorical, consistent with prior literature (Fainsilber & Ortony, 1987) and recent empirical findings (Pissani et al., 2025) showing that metaphors describing concrete topics (e.g., actions) are less appropriate than those describing abstract topics (e.g., emotions).

We observed several significant correlations in our norms. We found a moderate positive correlation between familiarity and frequency. These results, although not surprising, support the idea that the familiarity of an expression is a function of the frequency of its usage, which is in line with most of the literature comparing familiarity and different forms of frequency (e.g., Senaldi et al., 2022; Thibodeau & Durgin, 2011; Wisniewski & Murphy, 2005). We also found a moderate positive correlation between aptness and frequency, though it was weaker compared to the correlation between familiarity and frequency. This finding provides additional evidence that, unlike familiarity, aptness is not purely obtained by the repetition of an expression, but rather expresses the appropriateness—or quality—of a metaphor. We also found strong positive correlations between familiarity and aptness. We argue that, even though each variable expresses different dimensions of a metaphor, metaphors that are perceived to be more apt may be more frequently used in conversation and, therefore, those may become more familiar (Roncero & de Almeida, 2014; Thibodeau & Durgin, 2011).

Metaphors occur in various forms, including copular (e.g., *My lawyer is a shark*), verbal (e.g., *Maria devoured the paper*), and compound-like, two-word metaphor combinations (e.g., *brilliant idea*). However, most of the research to date has focused on the former. A major contribution of the present norming study is to endeavor beyond the *X is Y* structure. Two-word metaphor combinations represent particularly interesting cases for research because the relationship between the first and the second word of the expression is complex. First, two-word metaphor combinations include a variety of syntactic structures (i.e., adjective-noun and noun-noun). Second, they can carry the metaphorical content (or *vehicle*) in the modifier, in the head, or in both constituents. Third, the combination of both constituents can yield an expression that is meaningful only metaphorically or both metaphorically and literally. Together, the range of syntactic structures, vehicle position, and literalness can be informative of how metaphorical meaning is attained and, more broadly, how compositionality works in the mapping between linguistic expressions and the propositions that they express (see de Almeida & Lepore, 2018).

The present norms are a valuable resource for researchers interested in the nature of metaphor interpretation and semantic composition, with a focus on two-word metaphor combinations, rather than on the most typical copular forms. We provide a robust set of norms for studies requiring a large number of materials (e.g., those employing ERPs, fMRI, and computational modeling), while also allowing for their use in more restricted behavioral studies. Crucially, our materials are designed to investigate current theories of metaphor processing while considering integral factors known to affect the comprehension process.

## Footnotes

1 We would like to address an important issue, raised by an anonymous reviewer, on the similarities and differences between two kinds of expressions employed in the present set of norms: *compounds* and two-word *phrases*. Compounds are generally characterized by being syntactic units, which usually cannot be broken down; they have a particular stress pattern (compare *ladybug* with stress on “lady” [for a bug that is a lady] or on “bug” [for a type of insect, male or female]; see Gleitman & Gleitman, 1970, for a witty discussion). Our set contains both, expressions with words that co-occur to convey metaphorical content when they are phrases (*bright student*) but also when they are two-word compounds (*happy hour*). The similarities with lexicalized compounds (*heartbreak*) are that, as a syntactic unit, the phrase also has a modifier-head structure and can be further modified internally. Thus, a *miserable happy hour* still refers to *happy hour*, the compound, though it was not happy and may not have been an hour. But in* It’s often a happy* [*and joyful but sometimes somber…*] *hour for the family when Mary stops by* the reference is not necessarily to the *happy hour* of drinks and socializing, but more likely to a moment of joy, no matter what time of day. Most of our figurative expressions have the compound structure, though orthographically they are commonly written as two words. In this regard our norms differ from those of Gagne et al. (2019) whose compounds were purposefully chosen among those written with no spaces.

2 Most of the novel metaphors in Briner et al. (2018) were adapted from poetry (e.g., *impatient machinery, unkempt afternoon, fresh courage)* to ensure that participants were unlikely to have encountered them before*.*

3 Statistical note: Cumulative logistic regression estimates the probability of moving from one rating category to a higher one (e.g., from 1 → 2, from 2 → 3) rather than the magnitude of the increase. To make this more intuitive, we transformed the log odds for each condition into probabilities using the plogis function in R. The difference between these probabilities reflects the approximate increase (expressed as a percentage by multiplying by 100) in the likelihood of observing higher ratings when expressions are presented in one condition compared to another. All code and detailed steps are documented in the R script available in the OSF repository. For accessible introductions, see Winter (2019, Ch. 12) on logistic regression and Christensen (2024) on cumulative link mixed models.

## Declarations

### Availability of data and materials

Materials, data, and analysis code are available in the OSF repository at https://osf.io/xk3j9.
